# Developing a Label-Free Infrared Spectroscopic Analysis with Chemometrics and Computational Enhancement for Assessing Lupus Nephritis Activity

**DOI:** 10.3390/bios15010039

**Published:** 2025-01-11

**Authors:** Mei-Ching Yu, Xiang-Di Huang, Chin-Wei Kuo, Kai-Fu Zhang, Ping-Chung Liang, U-Ser Jeng, Pei-Yu Huang, Frederick Wai Keung Tam, Yao-Chang Lee

**Affiliations:** 1 Division of Pediatric Nephrology, Department of Pediatrics, Lin-Kou Chang Gung Memorial Hospital, Taoyuan 33302, Taiwan; tinapupss@gmail.com; 2 College of Medicine, Chang Gung University, Taoyuan 33302, Taiwan; 3 Department of Chemical Engineering, Ming Chi University of Technology, New Taipei City 243303, Taiwan; 4 National Synchrotron Radiation Research Center, Hsinchu 300092, Taiwan; cwkuo1216b@gmail.com (C.-W.K.); zjack5109@gmail.com (K.-F.Z.); steven1026309@gmail.com (P.-C.L.); usjeng@nsrrc.org.tw (U.-S.J.); pyhuang@nsrrc.org.tw (P.-Y.H.); 5 Centre for Inflammatory Disease, Department of Immunology and Inflammation, Imperial College London, London W12 0NN, UK; f.tam@imperial.ac.uk; 6 Department of Optics and Photonics, National Central University, Chung-Li 320317, Taiwan; 7 Department of Chemistry, National Tsing Hua University, Hsinchu 30044, Taiwan

**Keywords:** lupus nephritis, FTIR spectroscopy, IgG glycosylation, prognosis prediction function, relative absorption difference, iPath

## Abstract

Patterns of disease and therapeutic responses vary widely among patients with autoimmune glomerulonephritis. This study introduces groundbreaking personalized infrared (IR)-based diagnostics for real-time monitoring of disease status and treatment responses in lupus nephritis (LN). We have established a relative absorption difference (RAD) equation to assess characteristic spectral indices based on the temporal peak heights (PHs) of two characteristic serum absorption bands: ν_1_ as the target signal and ν_2_ as the PH reference for the ν_1_ absorption band, measured at each dehydration time (t) during dehydration. The RAD gap (Ψ), defined as the difference in the RAD values between the initial and final stages of serum dehydration, enables the measurement of serum levels of IgG glycosylation (ν_1_ (1030 cm^−1^), ν_2_ (1171 cm^−1^)), serum lactate (ν_1_ (1021 cm^−1^), ν_2_ (1171 cm^−1^)), serum hydrophobicity (ν_1_ (2930 cm^−1^), ν_2_ (2960 cm^−1^)), serum hydrophilicity (ν_1_ (1550 cm^−1^), ν_2_ (1650 cm^−1^)), and albumin (ν_1_ (1400 cm^−1^), ν_2_ (1450 cm^−1^)). Furthermore, this IR-based assay incorporates an innovative algorithm and our proprietary iPath software (ver. 1.0), which calculates the prognosis prediction function (PPF, Φ) from the RAD gaps of five spectral markers and correlates these with conventional clinical renal biomarkers. We propose that this algorithm-assisted, IR-based approach can augment the patient-centric care of LN patients, particularly by focusing on changes in serum IgG glycosylation.

## 1. Introduction

Systemic lupus erythematosus (SLE) is a chronic autoimmune disease causing inflammation and organ damage, particularly in the kidneys [1,2]. In particular, lupus nephritis (LN) is a severe SLE complication that is more prevalent in children (50–80%) than adults (20–40%) [3,4]. Despite medical advancements, childhood-onset LN (cLN) remains a leading cause of pediatric chronic kidney failure [5,6] and carries significant global mortality and morbidity [7,8], with nearly 44% of patients progressing to end-stage kidney disease (ESKD) within 15 years of LN onset [9].

LN involves complex immunoregulatory pathways, focusing on the non-specific activation of B lymphocytes. This leads to the deposition of circulating antigen-antibody immune complexes in the kidneys, contributing to the diverse types of LN [10,11]. Current immunosuppression (IS) regimens typically combine corticosteroids with cyclophosphamide, mycophenolate mofetil, or other biological agents, with treatment decisions guided by renal histology [10,12]. However, patients with similar LN histology may respond inconsistently, influenced by diverse immune pathways, ethnicity, and pharmacogenomics [13,14,15]. This inconsistency undermines the value of repeated kidney biopsies for managing the disease [16,17]. Furthermore, traditional biomarkers such as serum creatinine (Scr) and proteinuria lack sufficient precision for early detection of renal progression or outcomes in SLE [18,19]. An increase in Scr, for instance, may not be detected until up to 50% of renal function has already been lost. Additionally, the severity of proteinuria can vary significantly, especially in the advanced stages of nephritis. These limitations underscore the urgent need for more precise and reliable biomarkers to enable early detection and intervention in lupus patients at risk of LN progression.

Over the past two decades, growing research has shown the potential of attenuated total reflection Fourier transform infrared (ATR-FTIR) spectroscopy across various biomedical sciences by analyzing biofluids, tissues, and cells [20,21,22,23]. Despite its diverse applications, the use of ATR-FTIR in studying immune-mediated kidney diseases has recently been explored [22,23,24]. Overwhelmingly, this technology provides a sensitive, non-destructive approach that demands minimal sample preparation. Our previous studies have successfully utilized FTIR spectroscopy to analyze alterations in protein glycosylation associated with specific diseases and cancers [25,26,27,28]. In fact, sufficient evidence has revealed that variations in serum IgG glycosylation patterns and albumin levels are linked to several autoimmune diseases [29,30,31,32,33]. For instance, specific glycosylation patterns have been identified to influence the severity of disorders such as SLE and LN [34]. Serum IgG fucosylation has been correlated with active LN, affecting podocyte injuries [35].

In this study, we have developed a technique that addresses several critical limitations of current diagnostic methods. This novel approach is minimally invasive, cost-efficient, and capable of providing multiparametric data from a small blood sample in a single, rapid test. Using ATR-FTIR spectroscopy combined with our proprietary iPath software, we monitored the serial and temporal alterations in spectral marker absorption associated with serum biomolecules. Using the prognosis prediction function (PPF), our proposed method offers distinct advantages over existing diagnostic approaches for SLE and LN. These advantages include reduced sample requirements, improved efficiency, and comprehensive multiparametric analysis. The key spectroscopic features including hydrophobicity (Hp), hydrophilicity (Hph), albumin (Alb), lactate (Lact), Ig glycosylation (Gly), and the serum hydrophobicity index (HPI) were analyzed during controlled dehydration. We aim to develop a predictive model using ATR-FTIR to accurately forecast therapeutic responses, disease status (remission or LN flare-ups), and potential renal outcome prediction. This approach holds promise for advancing personalized medicine in managing autoimmune glomerulonephritis, especially for patients with LN.

## 2. Materials and Methods

### 2.1. Study Protocol and Patients

Eight SLE patients with biopsy-proven class IV LN, diagnosed at age ≤ 18 years (median age 16.3 years, female/male = 7:1), and six age-matched healthy young adults (median age 20.35 years, female/male = 3:3) were studied. Patients (P1–P8) were categorized based on their treatment: (1) P1–P4 with active cLN underwent standard induction IS (corticosteroid and cyclophosphamide or mycophenolate mofetil) within six months of diagnosis; (2) P5–P8 with chronic cLN received maintenance IS (azathioprine or mycophenolate mofetil). Additional treatments, such as calcineurin inhibitors and/or rituximab, were prescribed for non-responsive or renal flare-up cases.

A series of serum samples were collected from 2017 to 2020 during visits or hospitalization (Table S1), processed at 2500 rpm (4 °C) for 10 min, and stored at −80 °C before analysis. Medical records supplied demographic and laboratory details.

### 2.2. Statistical Analysis

Serum spectral data from acute (P1–P4) and chronic (P5–P8) cLN patients, presented as the median and the interquartile range (IQR), were analyzed using the Mann–Whitney test, with a significance level set at *p* < 0.05 (Origin Pro software 2023, Northampton, MA, USA).

### 2.3. Experimental Procedure and Equipment Setup

**Figure 1:**
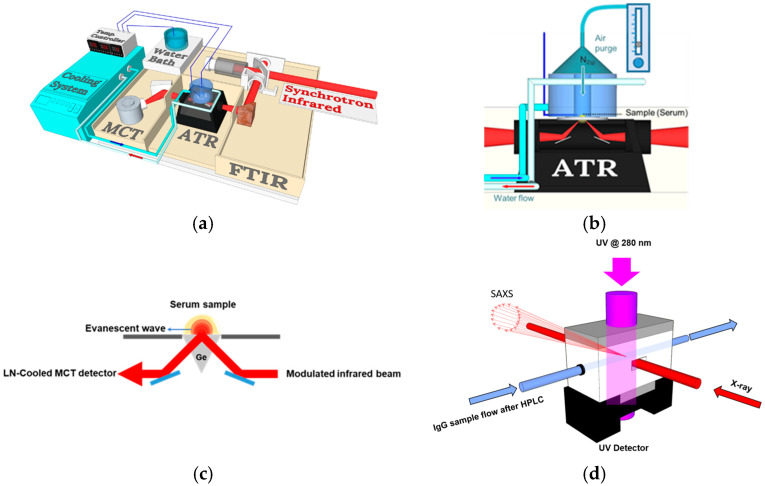
The schematic optical layout of the FTIR spectrometer, ATR accessory, and sample cell for the measurement of X-ray scattering. (**a**) The setup of the ATR-FTIR and temperature control systems for acquiring spectra of serum samples during dehydration. (**b**) The liquid serum sample loaded on the surface of the ATR germanium (Ge) crystal, which is mounted on a stainless steel disc and purged with a continuous flow of dry nitrogen. (**c**) The optical path of the evanescence wave of the modulated mid-IR beam as it propagates through the serum sample. (**d**) The sample cell is designed to collect small-angle X-ray scattering from IgG antibodies at TPS 13A1 of NSRRC. The IgG sample was purified using HPLC and detected by a UV detector to determine the IgG concentration.

ATR-FTIR spectroscopy was used to acquire consecutive 758 FTIR spectra per 0.791 sec for each serum sample during dehydration. The equipment setup comprised an FTIR spectrometer (Nicolet 6700, Waltham, MA, USA), an LN-cooled MCT detector, an ATR optical accessory, and a temperature-controlled system at TLS BL14A1 of NSRRC. FTIR spectra identified the IR absorption of serum components when dehydrated under dry nitrogen gas. Each spectral reading was obtained with two scans at a 4 cm^−1^ resolution. Frozen serum samples, pre-warmed to 3 °C from −80 °C, were prepared for ATR-FTIR measurement on a Ge crystal at 23 °C. Further details of the procedures are illustrated in Figure 1.

### 2.4. Determining Prognosis Prediction Function (PPF) Values in LN Patients: Analyzing Serum Spectral Indices Through Relative Absorption Difference (RAD)

Using state-of-the-art ATR-FTIR technology, we captured comprehensive insights into temporal shifts in serum spectral indices, such as hydrophobicity, hydrophilicity, lactate, albumin, and glycosylation (galactosylation and sialylation) of IgG antibodies during dehydration. Herein, we defined the temporal RAD as the relative change in the peak height (PH) of a spectral biomarker compared to the PH of a reference biomarker during serum dehydration, as shown in Equation (1). This ratio is calculated by taking the difference between two baseline-corrected PHs, **PH_1_**($\nu_{1}$, t, T) and **PH_2_**($\nu_{2}$, t, T), of the temporal IR absorption bands Abs_1_($\nu_{1}$, t, T) and Abs_2_($\nu_{2}$, t, T) for specific spectral indices, respectively, at the acquisition time (t) to **PH_1_**($\nu_{1}$, t, T) of the absorption band Abs_1_($\nu_{1}$, t, T) for serum samples collected during each patient follow-up (T). The peak height **PH_2_**($\nu_{2}$, t, T) is set as the absorption band reference to the peak height **PH_1_**($\nu_{1}$, t, T).

(1) $$RAD\left(\nu_{1},\nu_{2},t,T\right)=\frac{{PH}_{1}\left(\nu_{1},t,T\right)-{PH}_{2}\left(\nu_{2},t,T\right)}{{PH}_{1}\left(\nu_{1},t,T\right)}$$

PH_1_ and PH_2_: peak height of two spectral makers $\nu_{1}$ and $\nu_{2}$, respectively.

T and t: dehydration time of serum sample and follow-up frequency for a patient, respectively.

**Figure 2:**
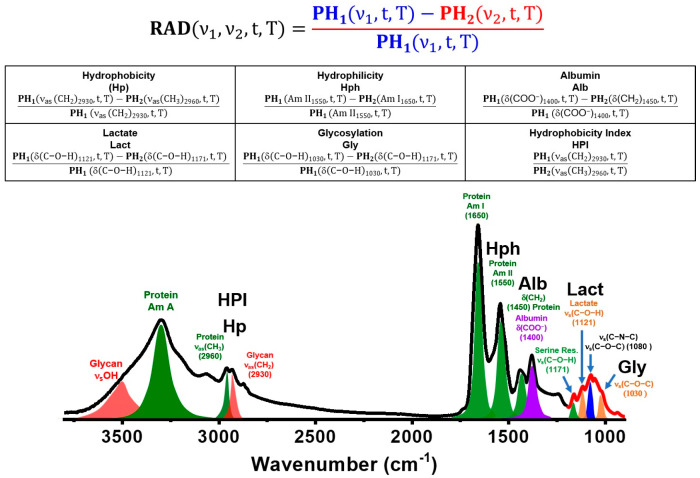
The RAD equation is defined by five spectral indices (**Lact**, **Gly**, **Hp**, **Hph**, and **Alb**) along with their corresponding band assignments.

As illustrated in Figure 2, the temporal RAD profiles of five specific spectral indices provide insights into the temporal absorption intensity of dried serum biocomponents: (1) hydrophobicity index (**Hp**, $\nu_{1}$ at 2929 cm^−1^ and the reference absorption band $\nu_{2}$ at 2960 cm^−1^); (2) hydrophilicity index (**Hph**, $\nu_{1}$ at 1546 cm^−1^ (amide II band) and the reference absorption band $\nu_{2}$ at 1650 cm^−1^ (Am I band); (3) albumin index (**Alb**, $\nu_{1}$ at 1400 cm^−1^ and the reference absorption band $\nu_{2}$ at 1450 cm^−1^); (4) lactate index (**Lact**, $\nu_{1}$ at 1121 cm^−1^ and the reference absorption band $\nu_{2}$ at 1171 cm^−1^); and (5) glycosylation index (**Gly**, $\nu_{1}$ at 1030 cm^−1^ and the reference absorption band $\nu_{2}$ at 1171 cm^−1^). The absorption band at 1171 cm^−1^, assigned to the C-O-H function groups found in serine, threonine, and tyrosine residues of proteins, serves as a reliable reference point for evaluating the **Gly** index values. Lipid-related molecules, such as triglycerides, cholesterol, and phospholipids, can introduce additional absorption intensity that affects the RAD values of **Gly** and **HPI**. Therefore, the RAD values are adjusted based on the standard absorption intensities of triglycerides to compensate for this interference during the temporal RAD calculation in this study. The iPath software also includes an optional function for the intensity calibration of triglycerides to eliminate interference.

**Figure 3:**
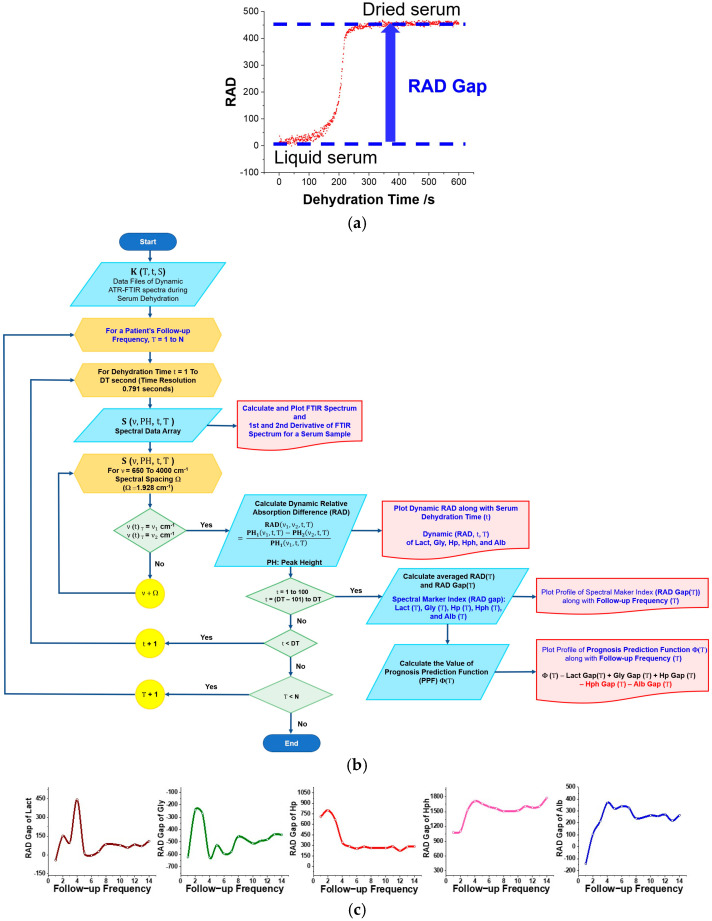
(**a**) A representative temporal RAD profile and RAD gap during serum dehydration. (**b**) Flowchart illustrating the data processing steps for temporal RAD and RAD gap determination during serum dehydration, and the calculation of PPF values for each follow-up visit. (**c**) Representative RAD gap profiles of **Lact**, **Gly**, **Hp**, **Hph**, and **Alb** of patient P1 throughout the study.

The rationale behind the RAD gap is to express the active concentration difference between the liquid and dehydrated phases of a spectral marker in a serum sample, as shown in Figure 3a. The gap value is calculated by subtracting the average RAD value of the final 100 measurements during the dehydration phase from the average RAD value of the initial 100 measurements in the liquid phase of a serum sample. This averaging process minimizes fluctuations, ensuring accurate and reliable data interpretation. The gap highlights the challenges hydrophilic proteins, such as albumin and IgG, encounter in overcoming hydrogen bonding with water molecules during dehydration. Notably, this metric is crucial for the PPF algorithm in this LN diagnostic technique.

We further developed the iPath software to facilitate the clinical calculation of these RAD gaps: (1) The **Hp** gap, defined as (**PH_1_**(2930 cm^−1^) − **PH_2_**(2960 cm^−1^))/**PH_1_**(2930 cm^−1^), assesses the additional methylene group (-CH_2_) arising from IgG glycosylation-related anti-inflammatory responses. (2) The **Hph** gap reflects the relationship of the hydrogen bonding interaction with amide I ν(C=O) and amide II (coupling of ν(C-N) and δ(C-N-H) and is considered to relate to the water affinity of the protein components. The Hap gap reveals the water affinity influenced by the structural alterations of serum component proteins. (3) The **Gly** gap, related to the glycosylation of IgG antibodies, can indicate the degree of IgG glycosylation induced by autoimmunity or inflammation. (4) The Lact gap is related to the effective concentration of lactate in serum based on the intensity alteration of the characteristic absorption band of δ(C-O-H) of lactate at 1121 cm^−1^ during the serum dehydration. An elevated lactate level in serum may suggest that the patient is experiencing hypoxia or reduced glucose levels. (5) The Alb gap is related to the effective concentration of albumin in serum based on the characteristic absorption band of the δ(COO^−^) residues of amino acids of albumin.

Figure 3b outlines the data processing steps in calculating the temporal RAD, determining the RAD gaps during serum dehydration, and computing the PPF values. Using iPath, we assessed the follow-up profiles of the spectral index gaps, including **Lact**, **Gly**, **Hp**, **Hph**, and **Alb**, to gauge personalized treatment responses in patients (Figure 3c).

### 2.5. Selection Criteria for Band References in Spectral-Based RAD Equations

**Figure 4:**
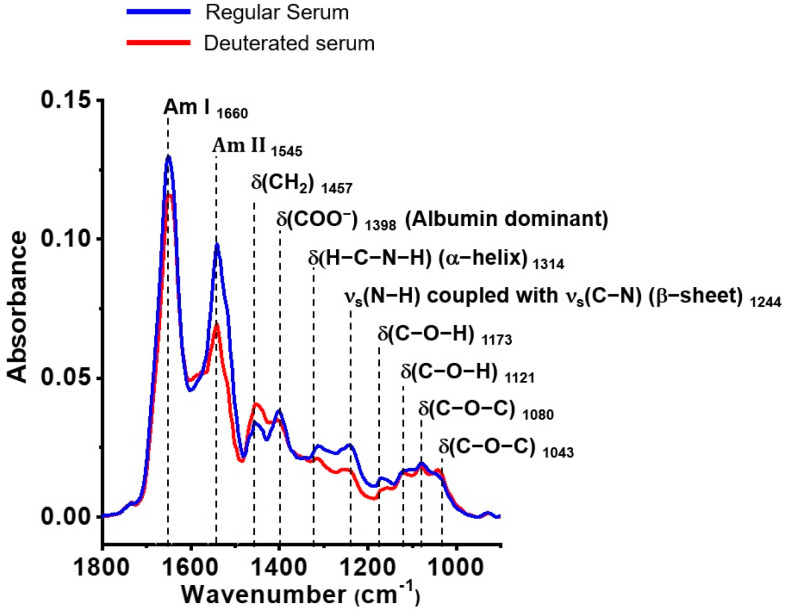
Representative FTIR spectrum and peak assignments of deuterated serum from a cLN patient (P6), demonstrating the stability of the amide I band peak height following a hydrogen–deuterium (H/D) exchange. Notably, the peak height of the amide I band of protein remains nearly unchanged post-H/D exchange.

In the RAD equation (see Equation (1)), the reference spectral intensities for the absorption bands should remain nearly unchanged or unaffected by the chemical modifications of the components and the absorption bands at nearby wavenumbers (Figure 4). This approach minimizes background fluctuations in the RAD temporal profile during serum dehydration. For each RAD equation, the intensity reference of the absorption band includes the methyl group ν_as_(CH_3_) at 2960 cm^−1^ for **Hp**, the amide I band at 1650 cm^−1^ for **Hph**, the methylene group δ(CH_2_) at 1450 cm^−1^ for Alb, and the δ(C-O-H) bands at 1171 cm^−1^ for Lact and Gly.

#### 2.5.1. Absorbance Reference for **Hp** in Human Serum

The reference absorption band for the TG-corrected PH is assigned to the methyl groups at 2960 cm^−1^, which predominantly contribute to serum proteins. The intensity of this band serves as a measure of the total protein content in the serum sample. It is used as the reference intensity in the RAD equation of **Hp** to enable the evaluation of alterations in the band intensity of the TG-corrected absorption PH of the methylene group ν_as_(CH_2_) at 2930 cm^−1^, corresponding to the glycan residues of IgG antibodies. The PH is associated with chemically adding methylene groups from galactose and sialic acid residues, a process catalyzed by glycosyltransferases during the glycosylation of Asn-297 glycan residues in IgG antibodies. This IgG glycosylation is hypothesized to occur in plasma B cells as part of the immune response.

#### 2.5.2. Absorption Reference for **Hph** in Human Serum

The amide I band, centered at 1650 cm^−1^, is critical for determining the protein secondary structure, as it reflects the hydrogen bonding framework of the amine (-NH) and carbonyl (C=O) groups in peptide bonds, providing structural stability under specific pH and temperature conditions. Thus, the amide I band is proposed as the intensity reference relative to the amide II band for **Hph**. As shown in Figure 4, the peak height of the amide I band remains nearly unchanged after H/D exchange in serum. However, a red shift in the amide II band is consistently observed, with the band center shifting from approximately 1545 cm^−1^ to 1457 cm^−1^. This shift is attributed to C-N-D bending vibrations in hydrophilic moieties of serum proteins, including albumin.

H/D exchange was performed by mixing 0.5 µL of serum sample with an equal volume of deuterated water (D_2_O), where deuterium (^2^H) replaces protium (^1^H). A more significant **Hph** gap indicates a higher concentration of hydrophilic proteins, suggesting a more significant dehydration barrier due to stronger hydrogen bonding between water and serum proteins. Conversely, a smaller **Hph** gap suggests weaker interactions. The **Hph** gap offers potential for monitoring variations in hydrophilicity caused by the glycosylation or glycation of IgG antibodies and albumin, which may contribute to immune-mediated diseases.

#### 2.5.3. The Absorption Reference for the Alb Index

The absorption bands at 1450 cm^−1^ (methylene group, δCH_2_) and 1400 cm^−1^ (primarily from carboxylic acid residues, δCOO^−^, and to a lesser extent from methyl groups, δCH₃) in serum are mainly associated with hydrophilic protein moieties, particularly albumin in healthy people. However, these bands are also partially influenced by methylene groups from lipid-related molecules, such as triglycerides, cholesterol, and phospholipids. In this study, the analysis incorporates a lipid correction for the RAD value of the Alb index to account for these contributions.

#### 2.5.4. The Absorption Reference for the Lact and Gly Indices

The Lact and Gly indices utilize the same reference absorption band at 1171 cm^−1^, attributed to serine or threonine residues in serum proteins. This reference band is located near the spectral biomarkers for Lact (1121 cm^−1^, characteristic of lactate absorption) and Gly (1030 cm^−1^, characteristic of glycosidic bonding absorption). The vibration frequency (or wavenumber) of this reference band remains consistent across all analyzed serum samples, making it a reliable reference point. It is used to evaluate intensity changes in the absorption bands at 1121 cm^−1^ and 1030 cm^−1^, which correspond to the lactate levels and IgG glycosylation, respectively.

### 2.6. PPF Algorithm for Patient-Centric Treatment Monitoring and Outcome Prediction

This study utilized consecutive ATR-FTIR serum spectra from cLN patients to integrate five RAD gaps with clinical parameters, including serum creatinine (Scr), serum albumin, urine total protein (UTP), and urine protein-to-creatinine ratio (UPCR). By combining these data, we established a personalized algorithm for each patient. This infrared (IR)-based method enables the dynamic monitoring of albumin and lactate levels, evaluation of serum protein hydrophobic and hydrophilic properties, and tracking of IgG modifications in response to nephritis activity or immunosuppressive (IS) treatment.

The temporal profiles of clinical laboratory parameters showed distinct correlations with RAD gap profiles: the **Hp** gap positively correlated with Scr, UTP, and UPCR but negatively correlated with serum albumin. In contrast, the **Hph** and **Alb** gaps displayed the opposite trends. Higher gaps for **Lact** and **Gly** were significant in lupus patients with active or acute cLN and were positively correlated with temporal changes in UPCR, UTP, and Scr, while they were negatively correlated with serum albumin. These findings suggest that elevated glycosylation or IgG and lactate levels, indicative of IgG chemical modifications, are likely driven by immune reactions associated with nephritis activity or treatment responses.

The **HPI** profile was developed to complement these observations. Serial analyses revealed a positive correlation between the **HPI** and clinical urine biomarkers (UPCR and UTP) and a strong correlation between the **HPI** and the serum **Hp** index. These results suggest that the **HPI** may be a viable alternative for assessing inflammation in LN patients.

Building on these findings, we introduced a prognosis prediction function (PPF) based on the linear combination of RAD gaps from each spectral marker, as illustrated in Equation (2). This PPF facilitates the rapid evaluation of treatment responses and nephritis status for individual cLN patients at each follow-up (T). Additionally, the normalized values of patient-specific Scr, serum albumin, UTP, and UPCR were combined linearly, as shown in Equation (3), providing a comprehensive framework for monitoring disease progression and therapeutic efficacy.

(2) $$\Phi_{PPF}\left(T\right)=\psi_{Lact\;Gap}\left(T\right)+\psi_{Gly\;Gap}\left(T\right)+\psi_{Hp\;Gap}\left(T\right)-\psi_{Hph\;Gap}\left(T\right)+\psi_{Alb\;Gap}\left(T\right)$$

Combined Normalized Biomarker Score = UTP + UPCR + Scr − Serum Albumin (3)

## 3. Results

### 3.1. Comparative FTIR Serum Spectra Analysis Between Proliferative cLN Patients and Healthy Controls

**Figure 5:**
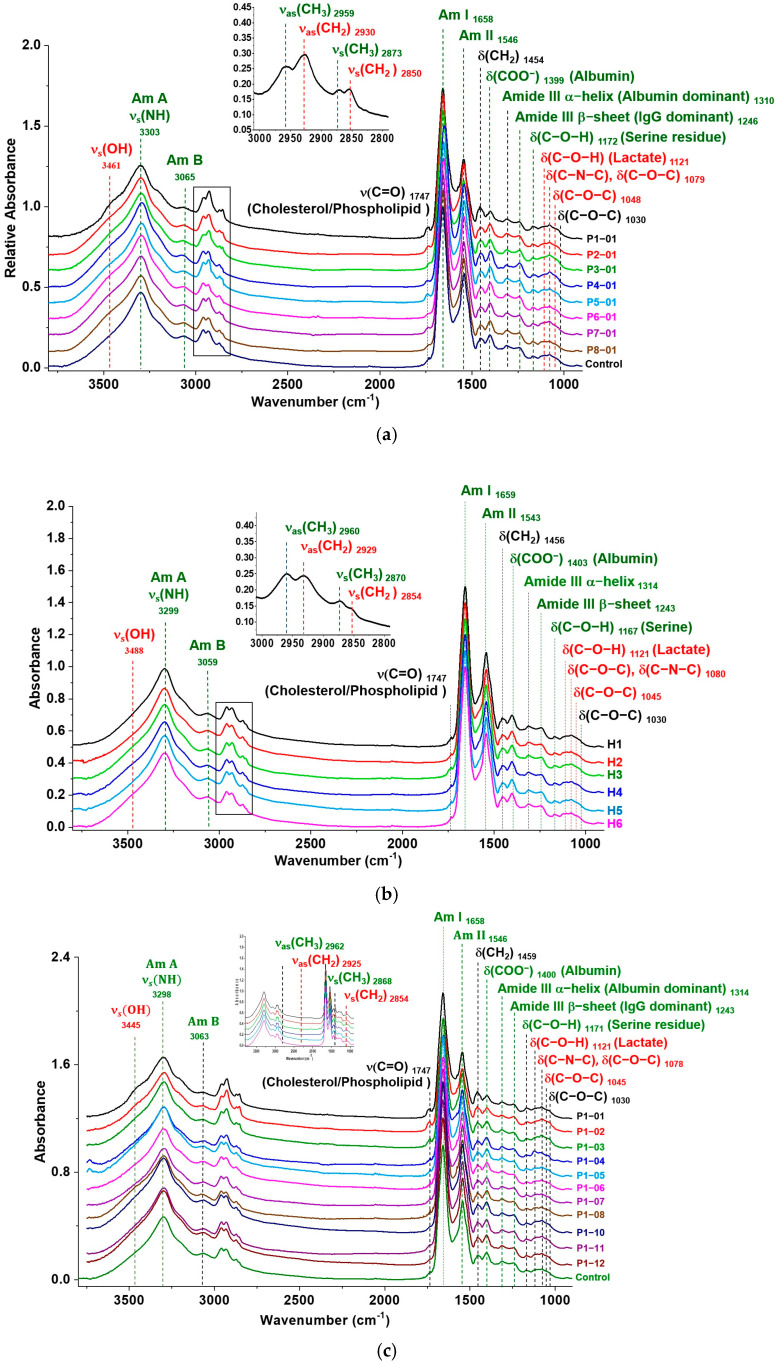
Representative FTIR spectra of dehydrated serum samples: (**a**) SLE patients with class IV LN (P1-01 to P8-01) and the averaged spectrum of 6 healthy cases as the control. (**b**) Healthy controls (H1-H6), and (**c**) serial serum spectra (P1-01 to P1-12) and the control illustrating changes from patient P1 diagnosed with acute cLN over the treatment period following the initial diagnosis. The red inset shows the assignment of the absorption peaks in the spectral range of 3000–2800 cm^−1^. Each serum spectrum underwent ATR correction, baseline correction, and normalization. (Note: P1-01 to P8-01 represent the first sample collected and analyzed after enrollment in the study; P1-01 to P1-12 present serum samples collected from patient P1 from day 0 to day 1191).

The FTIR spectra of dehydrated serum from eight proliferative (class IV) cLN patients (P1-01 to P8-01) and six healthy volunteers (H1–H6) are distinctly distinguishable (Figure 5a,b). The average follow-up during this study was 771 days, ranging from 338 to 1191 days. Among these, serial spectra were collected from patient P1 with cLN across the treatment period from diagnosis to day 1191 (Figure 5c). Fundamentally, these spectra reveal distinctive absorption patterns and the vibrational transitions of various serum components in the mid-IR spectral region (4000 cm^−1^ to 650 cm^−1^), such as lipids, albumin, and IgG antibodies.

Significant spectral differences were observed between cLN patients and controls in the range of 1200–900 cm^−1^, attributable to glycosylation and glycation. This spectral differentiation was mainly related to the absorption of the glycosidic bond C-O-C and glycated bond C-N-C and the C-O bond of monosaccharide residues of oligosaccharides covalently attached to IgG antibodies post-glycosylation.

In the range of 3000–2800 cm^−1^, the methylene (CH_2_) group is predominantly found in the hydrophobic moieties of molecules such as triglycerides, cholesterol, and glycan residues on serum IgG. Glycosylation and glycation can add more CH_2_ groups to serum proteins, attaching them to specific carbons in galactose (carbon #5) or sialic acid (carbons #3, #7, and #9) of glycans. Conversely, the methyl (CH_3_) group is primarily attributed to serum protein molecules, including albumin and IgG.

The spectral fingerprint of biocomponents is predominantly observed in the 1500–900 cm^−1^ range, where each component exhibits a distinct fingerprint, making ATR-FTIR technology invaluable for accurately identifying shifts in molecular composition. In the 1200–900 cm^−1^ range, IR absorptions reveal bending vibrations of C-O-C and C-O-H in oligosaccharide residues resulting from the glycosylation of IgG, lactate, and specific amino acids. Specifically, the absorption band at ~1171 cm^−1^ corresponds to the C-O of the C-O-H groups in serine, threonine, and tyrosine residues within proteins.

Furthermore, the absorption bands at 1121 cm^−1^ and 1045 cm^−1^ are assigned to lactate, a byproduct of anaerobic metabolism. Elevated serum lactate levels indicate tissue hypoxia or impaired oxygen delivery [36], associated with unfavorable outcomes in patients with acute kidney injury or chronic kidney disease [37,38]. Accordingly, our results demonstrate chemically modified protein components and metabolites resulting from glycosylation, glycation, and tissue hypoxia, while also highlighting alterations in serum physical properties such as hydrophobicity and hydrophilicity. These changes may correlate with the status of nephritis and treatment effectiveness. The assignments for significant absorption bands in serum are provided in Table 1.

### 3.2. Correlations Between FTIR Spectra Features and Ig Glycosylation Patterns

Lupus pathogenesis involves binding antibodies to various forms of DNA, such as anti-dsDNA and anti-ssDNA, and associated proteins [45,46]. Elevated levels of anti-dsDNA antibodies are closely associated with severe lupus activity, particularly affecting the kidneys. IgG antibodies consist of two central regions: an antigen-binding fragment (Fab) and a fragment crystallizable (Fc) region. After acquiring a necessary N-linked oligosaccharide chain, the latter binds to complement proteins and FcγR on effector cells. This glycan chain is further modified in the Golgi apparatus of plasma cells, where galactose, fucose, or sialic acid is added at asparagine 297 (Asn-297) in the Fc region’s CH2-84.4 domain, enhancing the IgG antibody’s affinity for its targets [29,47,48,49,50].

**Figure 6:**
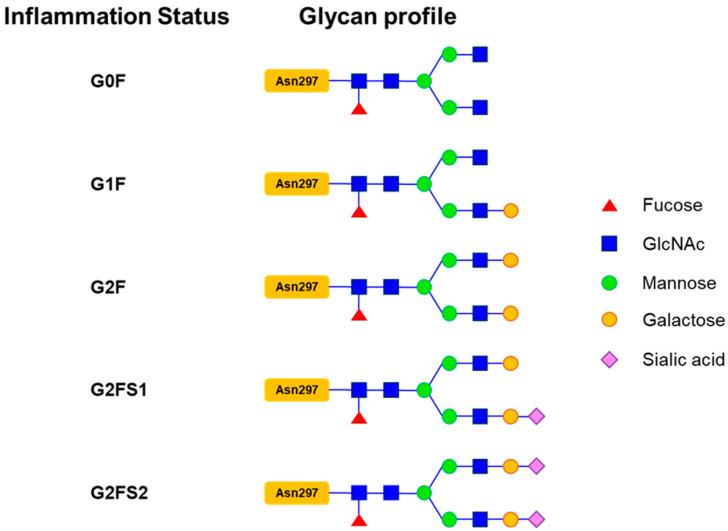
The schematic diagram illustrates the structure of the Asn-297 glycan residue of IgG and outlines the progression of glycosylation changes across various states of inflammation, transitioning from agalactosylated (G0F) to galactosylated (G1F, G2F), and subsequently to fucosylated and sialylated forms (G2FS1, G2FS2). These sequential modifications of the glycan structure highlight the dynamic shifts in agalactosylation, galactosylation, fucosylation, and sialylation.

Changes in IgG glycosylation during immune responses and inflammation result in altered N-glycan profiles [35]. As shown in Figure 6, typically, the most prevalent glycoform profile in healthy individuals is composed of galactosylated and afucosylated IgG N-glycans in the order of G1F > G2F > G2FS1, G2FS2 > G0F [35]. In LN, mass spectrometry has shown that only G0F was the predominant glycan profile in active disease [35]. Conversely, SLE patients without LN or those with nephritis in remission exhibited similar expressions of the G0F and G1F glycan profiles. Low levels of sialylated IgG glycans were consistently found across all lupus patients and healthy individuals, irrespective of LN status. Conclusively, in the initial phase of active LN, plasma B cells predominantly produce IgG with the G0F glycan—a less-glycosylated variant—influencing effector functions. IgG glycosylation evolves from galactosylation (G1F) to sialylation (G2FS1 or G2FS2) as inflammation progresses. These modifications are believed to aid immune complex clearance and reduce pro-inflammatory signaling. Sialyation enhances IgG’s affinity for anti-inflammatory receptors on immune cells and reduces pro-inflammatory cytokine production [47,51].

The FTIR fingerprint in the 1200–900 cm^−1^ range captures IgG glycosylation across different inflammation stages (Figure 5). For example, absorption bands for the anionic phosphodioxy group (PO^2−^) of total RNA were observed at ~1245 cm^−1^ (ν_as_ (PO^2−^)), ~1080 cm^−1^ (ν_s_ (PO^2−^)), and ~961 cm^−1^ (C-O RNA ribose chain), with peak height ratios of about 4:5:2. In lupus, distinct absorption bands at approximately 1245 cm^−1^ and 1080 cm^−1^ correspond to the characteristic absorption patterns of antibodies [52,53]. Interestingly, the cLN serum spectra lack an absorption band at 961 cm^−1^, indicating a minimal contribution from the PO^2−^ groups of total RNA. These observations suggest that cLN patient serum spectra predominantly originate from IgG glycan residues rather than RNA. Additionally, considering that the serum RNA concentrations are relatively low (1.4–0.35 ng) in our experiments, their impact on the FTIR spectra is minimal, further emphasizing the role of IgG glycans over RNA [53,54]. After a H/D exchange reaction, the absorption intensity at 1315 cm^−1^ and 1245 cm^−1^ decreased significantly (Figure 4), while the 1080 cm^−1^ band remained unchanged. This observation suggests a weak or non-existent relationship between the 1080 cm^−1^ band and serum RNA. These observations assume that cLN patient serum spectra predominantly originate from IgG glycan residues rather than RNA.

### 3.3. Analysis of Ig Glycosylation Profiles: Serum Deglycosylation and Fast Protein Liquid Chromatography (FPLC)-FTIR Spectral Study

In our analysis of consecutive serum spectra from acute cLN patient (P1) during IS treatment, we observed increased absorption of IgG glycan residues in follow-up samples P1–2 and P1–3, collected 1 and 3 months after nephritis diagnosis, respectively. P1 serum samples were deglycosylated with PNG_ase_ F, which cleaves the C-N-C bond linking N-linked glycans to Asn-297 on IgG, to investigate the spectral contribution of IgG glycans. After PNG_ase_ F treatment, the serum was separated by FPLC without phosphate.

**Figure 7:**
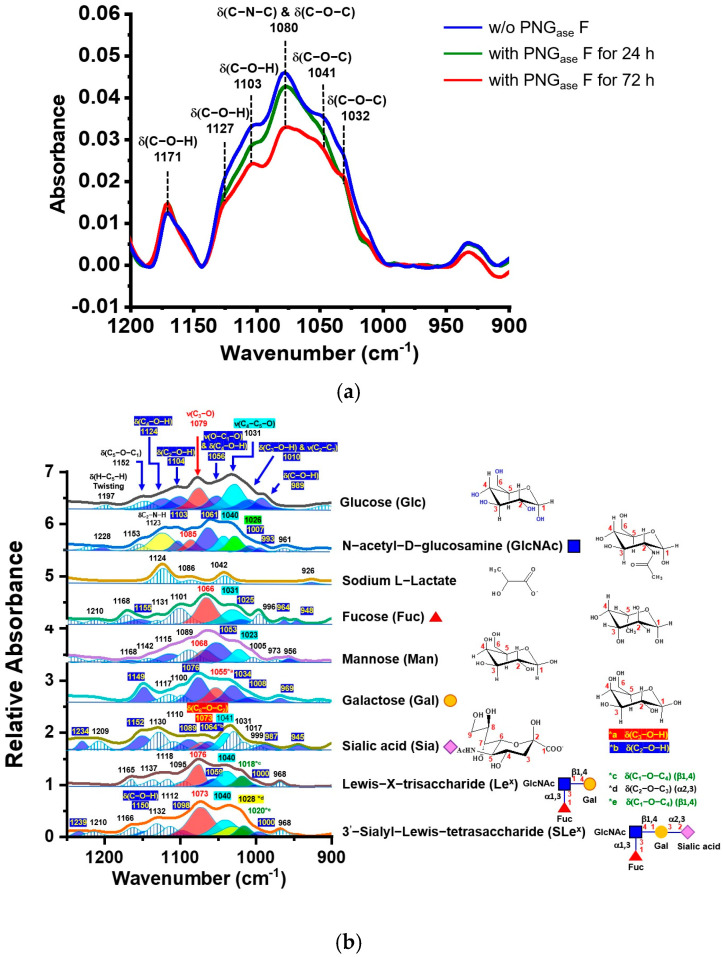
(**a**) ATR-FTIR spectra of serum samples (P1-8) treated with PNG_ase_ F for 24 to 72 h. (**b**) Representative serum ATR-FTIR spectra of selected monosaccharides, oligosaccharides, and monosaccharide derivatives in the 1300–900 cm^−1^ spectral range.

We noted reduced absorption at 1080 cm^−1^ in the treated serum compared to the untreated serum, indicating the presence of the C-N-C bond formed through condensing the amine of Asn-297 and the hydroxyl residue of IgG glycans (Figure 7a). Thus, our results confirm that the absorption band at approximately 1080 cm^−1^ primarily originates from the glycan bonding to Asn-297 of IgG. Moreover, the FTIR spectra revealed selected saccharides—such as glucose, N-acetyl-D-galactosamine (GalNAc), N-acetyl-D-glucosamine (GlcNAc), fucose, galactose, sialic acid, Lewis-X-trisaccharide (Le^x^), and sialyl-Lewis-X-tetrasaccharide (SLe^x^)]—along with sodium L-lactate, within the 1200–900 cm^−1^ fingerprint region (Figure 7b).

IgG sialylation accompanies a distinct spectral absorption at ~1028 cm^−1^, attributed to the glycosidic bond (α2,3) between Sia and Gal in Le^x^ and SLe^x^. This spectral shift arises from a condensation reaction in which the sialic acid interacts with the hydroxyl group of the GlcNAc residue in Le^x^, forming a glycosidic bond (C-O-C). An additional absorption band was observed at approximately 1018 cm^−1^, indicating the formation of a glycosidic bond between Gal and GlcNAc. Another absorption at ~1040 cm^−1^ was suggested to correspond to the stretching vibration motion of ν(C_2_-O) coupled with ν(C_4_-O) of the six-member ring of residues of Fuc, GlcNAc, and Gal in Le^x^. Accordingly, we suggest that absorptions in the range of 1040–1018 cm^−1^ indicate the formation of glycosidic bonds due to galactosylation or further sialylation in IgG. We utilize the absorption peak height at 1030 cm^−1^ to estimate the IgG glycosylation levels, thereby minimizing interference from blood sugar absorption.

Thus, we recommend using the distinctive absorption bands in the 1200–900 cm^−1^ fingerprint region for determining IgG glycosylation levels, encompassing fucosylation (G0F), galactosylation (G1F and G2F), and sialylation (G2FS1 and G2FS2). Monitoring the absorption intensity change of the glycosidic bond at 1030 cm^−1^ allows us to assess the altered N-glycan residues in IgG, suggesting that the N-glycosylated Fc regions of IgG antibodies could serve as a potential biomarker for diagnosing and monitoring autoimmune diseases such as SLE and LN.

### 3.4. FTIR Spectral Analysis of Human Serum Proteins: Albumin Spectral Index and Its Association with cLN Activity

SLE patients experiencing flare-ups or active LN commonly show decreased serum albumin levels. Hypoalbuminemia may indicate renal involvement in lupus and reflect disease activity. Our study revealed that ATR-FTIR spectra of dehydrated serum from lupus patients with active cLN at the onset of their treatment exhibited a reduction in absorption at ~1450 cm^−1^, closely related to the absorption intensity at 1400 cm^−1^ (Figure 5c).

Human serum albumin, comprising 585 amino acid residues, contains numerous specific amino acids, such as Lys9, His16, Arg24, Asp53, and others [55]. Approximately 23% are Asp and Glu, while 34% are Thr, Ala, Val, Ile, and Leu. The broad absorption of *δ*CH_2_ at ~1450 cm^−1^ is primarily attributed to the hydrophobic moieties of proteins. In contrast, the absorption band at ~1400 cm^−1^ predominantly corresponds to protein amino acid residues, especially the δ(COO^−^) absorption of the carboxylate group in Asp and Glu and, to a lesser extent, the δ(CH_3_) absorption of methyl groups in hydrophobic protein regions.

Given the chemical structure of albumin, it exhibits stronger absorption for δ(COO^−^) at ~1400 cm^−1^ compared to δ(CH_2_) at 1450 cm^−1^ [40,41]. As a result, the PH ratio of the absorption at 1400 cm^−1^ to that of 1450 cm^−1^ serves as a valuable indicator for assessing serum albumin levels. The IR-based albumin marker, PH (1400 cm^−1)^/PH (1450 cm^−1^), shows a positive correlation with the clinical albumin concentration measured in hospital laboratories.

Furthermore, the absorption in the spectral range of 1350–1200 cm^−1^ is assigned to the amide III band of serum protein, reflecting protein secondary structures. The absorption bands at approximately 1315 cm^−1^ and 1240 cm^−1^ are attributed to N-H in-plane bending coupled with C-N stretching, indicative of the protein secondary β-sheet structure, and the rocking vibration of the H-N(C=O) C-H fragment of the α-helix structure within the peptide framework [42,43,44].

### 3.5. Serum Hydrophobicity Index (**HPI**) and cLN Outcome: Remission or Flare

In our study, we utilized the absorption intensity ratio of the methylene to methyl groups in the range of 3000–2800 cm^−1^ to evaluate alterations in serum hydrophobicity resulting from IgG glycosylation after eliminating the absorption intensity induced by lipid-related species. This led to the proposal of the serum **HPI**, represented by the formula **PH_1_**(ν_as_(CH_2_))/**PH_2_**(ν_as_(CH_3_)), as a promising indicator for assessing the magnitude of IgG glycosylation in serum.

**Figure 8:**
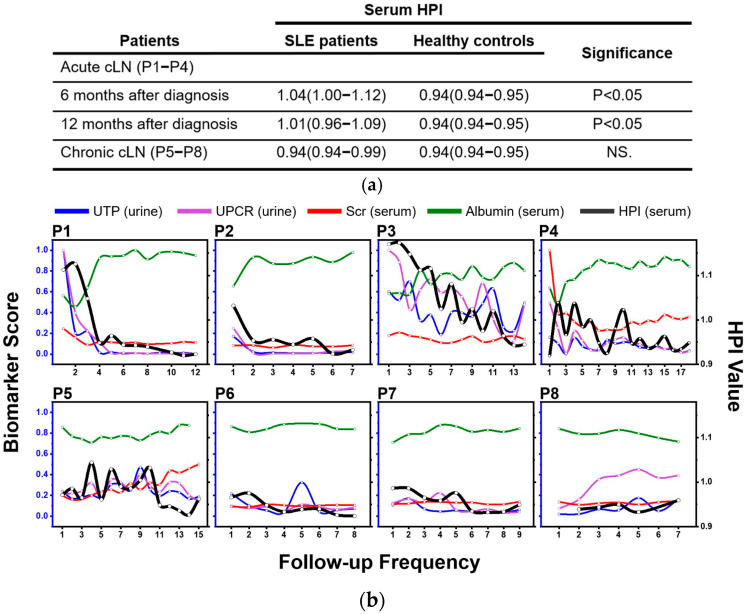
(**a**) Comparison of serum **HPI** levels among the patients with acute cLN, chronic cLN, and healthy control subjects. (**b**) Correlations between serum **HPI** values and clinical biomarkers in cLN treatment response. This figure illustrates the relationships of serum **HPI** with Scr, serum albumin, UTP, and UPCR in cLN patients throughout the study period. Patients P1–P4, with active cLN undergoing induction immunosuppression therapy, and patients P5–P8, with chronic cLN receiving maintenance immunosuppression therapy, are included. (For concentrations of individual clinical biomarkers at each follow-up time point for patients P1–P4 and P5–P8, refer to Table S1).

Additionally, serum **HPI** levels at the 6-month and 12-month intervals after an acute cLN diagnosis in patients P1–P4 were significantly higher than those in healthy controls (Figure 8a). Specifically, the value for acute cLN at 6 months was 1.04 (IQR 1–1.12), and at 12 months was 1.01 (IQR 0.96–1.09), compared to healthy controls at 0.94 (IQR 0.94–0.95), with a *p*-value of <0.05 (Mann–Whitney test). Concurrently, these patients exhibited a decline in blood albumin levels, corresponding to their response to medications such as prednisolone and Myfortic^®^, with or without Tacrolimus^®^. As nephritis achieved complete or partial remission, the serum **HPI** values approached 0.94 (IQR 0.92–0.94), similar to healthy controls (0.95, IQR 0.94–0.96).

In contrast, chronic cLN patients (P5–P8) undergoing a mycophenolate mofetil-based maintenance IS regimen displayed steady serum **HPI** values of 0.95 (IQR 0.94–0.99), similar to healthy controls. This consistency was observed regardless of the intensity of immunosuppression or additional biological agents (Figure 8b), even as these patients continued to exhibit proteinuria or impaired renal function. In conclusion, our findings indicate that regular, individualized monitoring of serum **HPI** and its fluctuations during treatment could provide a straightforward diagnostic approach for assessing treatment response, disease progression, and renal outcomes.

Furthermore, we also observed a positive correlation between the serum **HPI** and clinical renal markers, including Scr, UTP, and UPCR, in acute cLN patients (P1 to P4) (Figure 8). Conversely, a negative relationship was noted between serum HPI and serum albumin. Overall, serum **HPI** values in acute cLN increased significantly with disease severity, whereas in patients with chronic cLN, these values remained stable throughout the study period.

### 3.6. PPF and RAD Gaps: Assessing LN Activity, Treatment Response, and Clinical Biomarker Correlations

**Figure 9:**
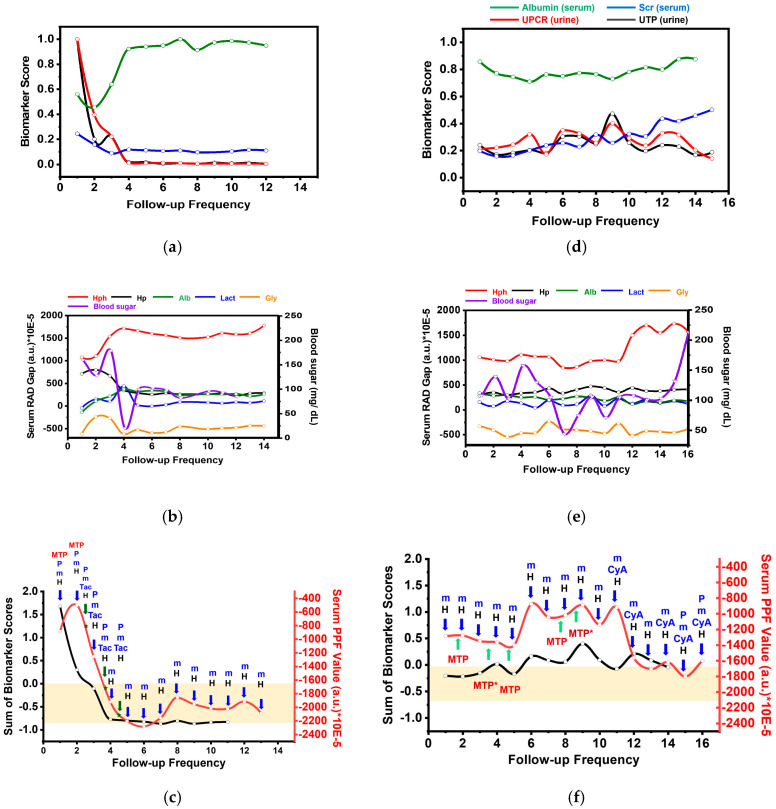
The treatment response of acute cLN patient P1 and chronic cLN patient P5. Through follow-up periods. (**a**,**d**) Biomarker scores, including albumin, Scr, UPCR, and UTP; (**b**,**e**) serum RAD gaps; and (**c**,**f**) PPF values. Abbreviations: MTP (Mini-pulse methylprednisolone), P (Prednisolone), m (Myfortic), Tac (Tacrolimus), CyA (Cyclosporin), H (Hydroxychloroquine).

The comparison of RAD gaps (**Hp**, **Hph**, **Lact**, **Alb**, and **Gly**) and clinical biomarkers (UTP, UPCR, Scr, and albumin) between an acute cLN patient (P1) and a chronic cLN patient (P5) revealed dynamic relationships reflecting disease activity and treatment response (Figure 9). Initially, P1 exhibited elevated Scr, UPCR, and UTP levels alongside low serum albumin (Figure 9a). Concurrently, serum spectral indices such as **Hp**, **Gly**, and **Lact** increased while the **Hph** gap decreased (Figure 9b). Following successful induction IS for acute cLN, P1’s Scr, UPCR, and UTP levels declined, showing a positive correlation with changes in the **Hp**, **Gly**, and **Lact** gaps and an inverse correlation with the **Hph** gap. A significant positive correlation was also observed between the clinical albumin concentration and the IR-based albumin index. The PPF, representing the aggregated RAD gaps, also consistently aligned with the clinical biomarker score, mirroring treatment progression and remission states (Figure 9c).

Furthermore, patient P1’s PPF positively correlated with the biomarker score during treatment, aligning with the levels observed in healthy individuals post-cLN remission. Outcomes for other active cLN patients (P2–P4) are presented in the Supplementary Materials (Figure S1a–i). Notably, P1’s hyperglycemia (160–179 mg/dL) corresponded with the Gly gap and the IgG glycosylation score (Figure S2a). This glycosylation score of isolated IgG, free from glucose interference, was identified within the 1200–1000 cm^−1^ IR spectral range (Figure S2b,c).

For further validation, we calculated the radius of gyration (Rg)—the average squared distance from the center of mass in IgG of patient P1, based on the Guinier region of the small angle X-ray scattering (SAXS) curve, acquired at the BioSAXS endstation TPS 13A at NSRRC in Taiwan [56]. The Rg results from the isolated IgG SAXS revealed higher values for initial serum samples P1-2 (57.4 ± 1.2 Å) and P1-3 (53.0 ± 0.4 Å), collected at the onset of acute LN diagnosis, compared to the follow-up sample P1-11 (50.0 ± 0.1 Å) and the healthy control (50.0 ± 0.3 Å for H6). This indicates that a higher Rg value of IgG is associated with acute inflammation, which may induce IgG glycosylation. Eventually, our findings suggest that an increased Gly gap could be attributed to additional galactosylation or sialylation of IgG during acute inflammation.

Furthermore, the clinical serum lactate profiles consistently matched the Lact index from iPath, affirming the accuracy of the Lact and Alb profiles as determined by the RAD gap (Figure S3). In contrast, chronic cLN patient P5 experienced a gradual decline in the estimated glomerular filtration (eGFR) to 65 µL/min/1.73 m^2^ and sustained proteinuria (UPCR > 2) despite normal serum albumin levels (Figure 9d). Moreover, her Alb gap corresponded with serum albumin concentrations. Her **Hp** and **Gly** spectral indices remained constant, but the spectral index stabilized until her eGFR dropped below 40 µL/min/1.73 m^2^. Unlike acute LN patient P1, P5 exhibited fluctuating lactate levels, and the PPF showed a positive correlation with the clinical biomarker score, highlighting its potential for monitoring therapy (Figure 9e,f). For instance, additional corticosteroid treatment for cLN decreased the PPF values despite negligible changes in the clinical biomarker score. Our findings suggest that, despite varying IS treatments for chronic cLN, their clinical biomarker scores and serum PPF values showed no significant changes. Outcomes for other cLN patients (P6–P8) are provided in the Supplementary Materials (Figure S1j–r).

## 4. Discussion

This study highlights the RAD gap as a valuable diagnostic tool for capturing dynamic changes in disease activity and treatment responses in SLE patients with cLN. By integrating five serum spectral indices—**Hp**, **Gly**, **Lact**, **Alb**, and **Hph**—the RAD gap effectively correlates with key clinical biomarkers such as Scr, UPCR, serum albumin, and lactate, reflecting biochemical changes during cLN progression and remission. While individual RAD gaps provide detailed insights into specific biochemical processes, their correlation with clinical biomarkers may vary based on the patient’s disease state and metabolic profile. To address this variability, we developed an IR-based immunoassay and a custom algorithm to derive prognosis prediction function (PPF) values, which aggregate the RAD gaps. The PPF demonstrated consistent alignment with clinical biomarker scores across diverse patient cases and showed greater sensitivity for therapeutic monitoring compared to traditional methods. These findings emphasize the potential of the RAD gaps and PPF to enhance personalized medical approaches and real-time monitoring of disease activity, improving the precision of clinical management for lupus nephritis. Integrating this approach into routine diagnostics could enable more effective and individualized treatment strategies.

Our research provides insight into serum spectral assignments, particularly IgG glycosylation. IgG glycosylation can increase the number of methylene groups (~2930 cm^−1^) in human serum through covalent modification by adding saccharides to glycan residues (fucosylation, galactosylation, and sialylation). These immunologically driven modifications enhance the absorption intensity of methylene groups, suggesting that the ratio of methylene to methyl group absorption intensities could serve as a potential indicator for assessing serum hydrophobicity and distinguishing LN patients from healthy individuals.

Integrating ATR-FTIR technology with the iPath software to facilitate clinical application and translation presents an innovative approach for managing LN, particularly focusing on monitoring treatment and prognostication through changes in serum IgG glycosylation.

## 5. Conclusions

This study introduces an innovative methodology using IR spectroscopy technology to assess LN. By integrating this technology with the existing spectral biology database, our findings demonstrate its potential as a diagnostic tool for evaluating the severity of SLE/LN. Nonetheless, several limitations necessitate further exploration. Future steps include increasing the sample size and ensuring broader diversity in patient demographics to enhance the generalizability of our results. Extending the duration of longitudinal studies will provide deeper insights into the long-term effectiveness and reliability of this approach.

Comparative analyses with other immunological biomarkers and analytical tools, such as mass spectroscopy, are essential to investigate its efficacy and accuracy further. Expanding our research in these directions is crucial for advancing clinical validation and enabling more expansive application of this technology in diagnosing and managing autoimmune kidney diseases, such as LN.

## Figures and Tables

**Table 1:** Assignments of characteristic absorption bands for biocomponents in human serum.

Wavenumber/cm^−1^	Spectral Assignment	Reference
**3290**	Amide A (N−H stretching vibration)	[21,27,39]
**3064**	Amide B (overtone of amide II)	[21,27,39]
**2958**	ν_as_(CH_3_) (CH_3_ antisymmetric stretching vibration, dominant contribution from proteins)	[21,27,39]
**2931**	ν_as_(CH_2_) (CH_2_ antisymmetric stretching vibration, dominant contribution from lipids)	[21,27,39]
**2872**	ν_s_(CH_3_) (CH_3_ symmetric stretching vibration, dominant contribution from proteins)	[21,27,39]
**2855**	ν_s_(CH_2_) (CH_2_ symmetric stretching vibration, dominant contribution from lipids)	[21,27,39]
**1737**	ν_s_(C=O) (cholesterol/phospholipid)	[27,39]
**1651**	Amide I (C=O stretching vibration, proteins)	[21,27,39]
**1541**	Amide II (vibration motion-coupled C−N stretching vibration and C−N−H bending vibration)	[21,27,39]
**1456**	δ_as_(CH_2_) (CH_2_ antisymmetric bending, scissoring, lipids and proteins)	[21,27,39]
**1399**	δ(COO−), δ_as_ (CH_3_) (COO^−^ dominant, −CH_3_ antisymmetric bending, lipids and proteins)	[21,27,39]
**1314**	Amide III, δ(H−C−N−H) (α-helix)	[40,41,42,43,44]
**1243**	Amide III, ν_s_ (N−H) coupled with ν_s_ (C−N) (β-sheet)	[40,41,43]
**1171**	δ(C−O−H) (predominantly contributed to bending vibration of serine residues of proteins)	[39]
**1122**	δ(C−O−H) (predominantly contributed to bending vibration of lactate in serum)	[39], this work
**1078**	δ(C−O−C) & δ(C−N−C) (bending of Asparagine-linked glycan and carbohydrates)	[39], this work
**1030**	δ(C−O−C) (bending vibration of carbohydrate)	[39]

## Data Availability

The original contributions presented in the study are included in the article and Supplementary Materials; further inquiries can be directed to the corresponding authors.

## Supplementary Materials

The following supporting information can be downloaded at: https://www.mdpi.com/article/10.3390/bios15010039/s1, Table S1: Concentrations of clinical biomarkers (UTP, UPCR, Scr, eGFR, serum albumin) for acute cLN patients P1–P4 and chronic cLN P5-P8 at each follow-up; Figure S1: Treatment response profiles in acute cLN patients (P2, P3, and P4) and cLN patients (P6, P7, and P8). Biomarker scores (i.e., of albumin, Scr, UPCR, and UTP) are presented for acute cLN patients in Panels (a–c) and for chronic cLN patients in Panels (j–l). Serum RAD gaps (i.e., **Hph**, **Hp**, **Alb**, **Lact**, and **Gly**) are shown for acute cLN patients in Panels (d–f) and for chronic cLN patients in Panels (m–o). PPF values alongside cumulative biomarker scores assessed at each follow-up during the study are displayed for acute cLN patients in Panels (g–i) and for chronic cLN patients in Panels (p–r); Figure S2: Correlation between IgG glycosylation and the **Gly** spectral index. Panel (a) illustrates blood sugar levels (black), Gly spectral index (blue), and IgG glycosylation levels (red) from lupus patient P1 with acute cLN. Panels (b,c) display glycosylation scores of IgG from serial serum samples of patient P1 and healthy controls (H1–H6), respectively, determined via FPLC. Figure S3: The figures present the correlation between the **Lact** spectral index and serum lactate levels throughout the treatment of cLN patients P3–P8.
