# Supplementary material for: Developing a Label-Free Infrared Spectroscopic Analysis with Chemometrics and Computational Enhancement for Assessing Lupus Nephritis Activity
Source: Biosensors (Basel). 2025 Jan 11;15(1):39. doi: 10.3390/bios15010039 (PMC11763532; doi:10.3390/bios15010039)
Supplement: Supplementary file 1 [file biosensors-15-00039-s001.zip › Figure S1.pdf]

Acute cLN patients

(a)

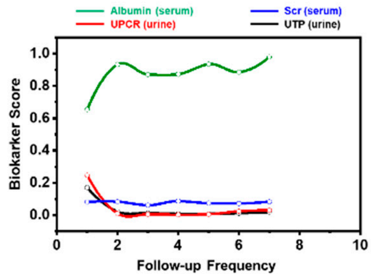

(b)

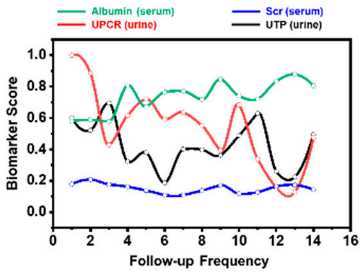

(c)

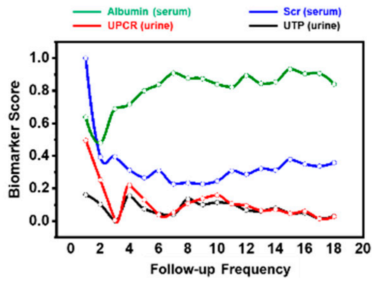

(d)

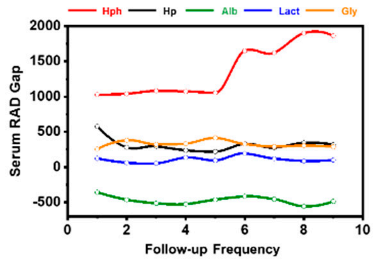

(e)

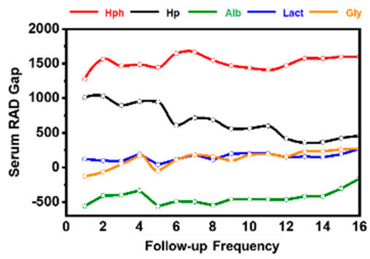

(f)

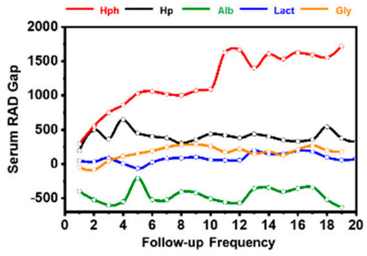

(g)

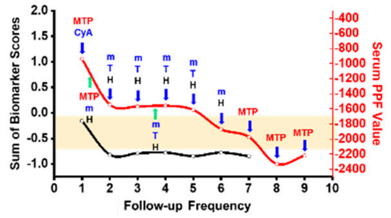

(h)

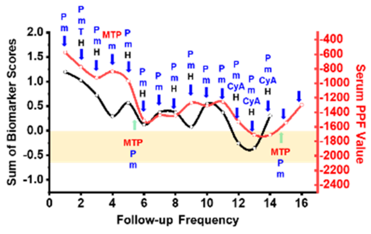

(i)

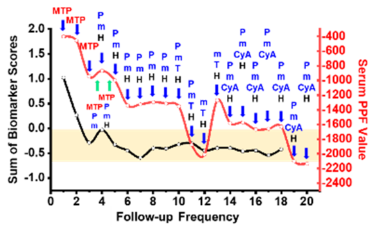

Chronic cLN patients

(j)

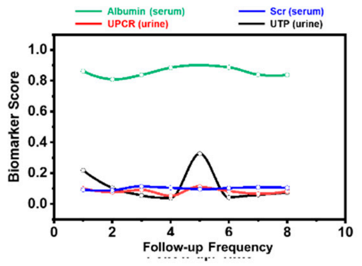

(k)

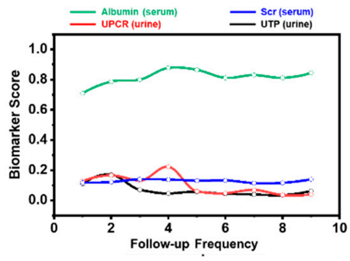

(l)

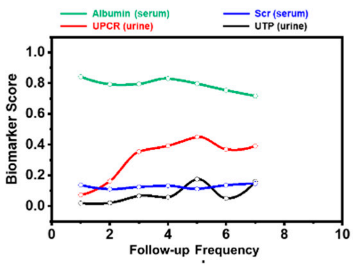

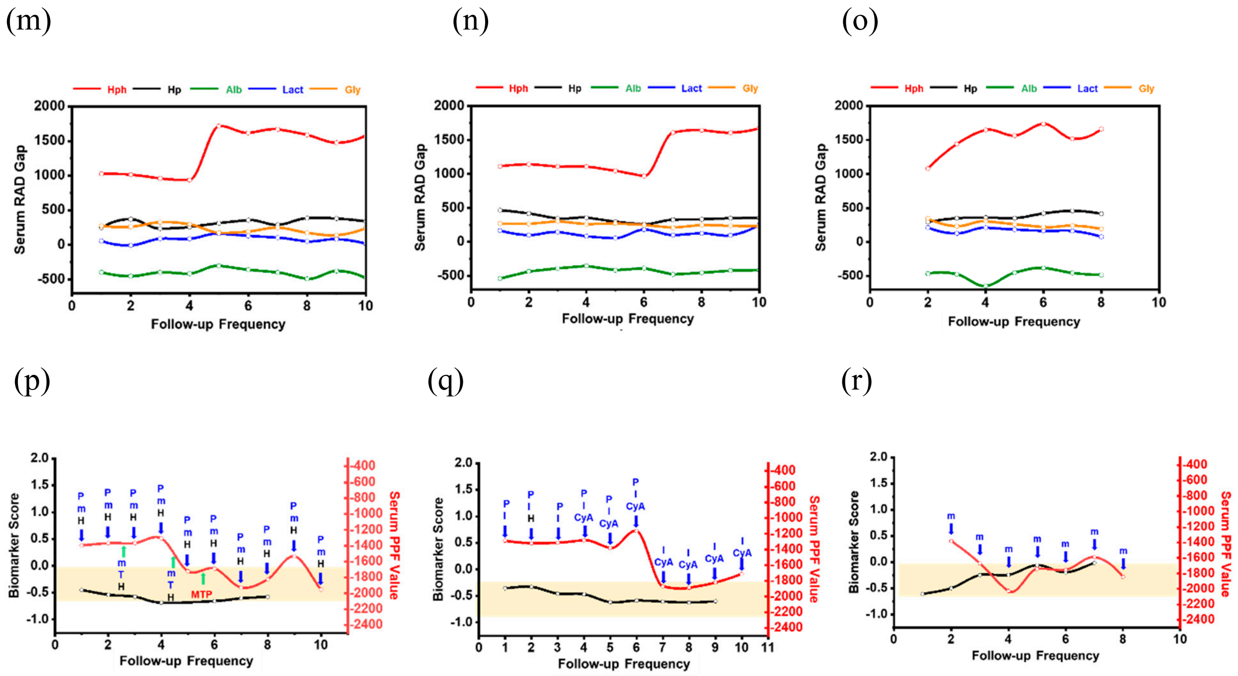

Figure S1. Treatment response profiles in acute cLN patients (P2, P3, and P4) and cLN patients (P6, P7 and P8). These figures display the treatment responses in individual patients through various clinical biomarkers and serum IR prognostic indices over the course of the study. Biomarker scores, including levels of albumin, Scr, UPCR, and UTP, are presented for acute cLN patients in **Panels (a, b, c)** and for chronic cLN patients in **Panels (j, k, l)**. Serum RAD gaps, referring to spectral marker indices of hydrophilicity (Hph), hydrophobicity (Hp), albumin (Alb), lactate (Lact), and Ig glycosylation (Gly), are shown for acute cLN patients in **Panels (d, e, f)** and for chronic cLN patients in **Panels (m, n, o)**. Prognosis Prediction Function (PPF) values, alongside cumulative biomarker scores assessed at each follow-up during the study , are displayed for acute cLN patients in **Panels (g, h, i)** and for chronic cLN patients in **Panels (p, q, r)**. Treatment abbreviations include MTP (Mini-pulse methylprednisolone), P (Prednisolone), m (Myfortic), I (Immufine), Tac (Tacrolimus), A (Azathioprine), CyA (Cyclosporin), H (Hydroxychloroquine), L (Losartan), N (Nifedipine), La (Labetalol), R (Rosuvastatin)
