# Supplementary material for: Developing a Label-Free Infrared Spectroscopic Analysis with Chemometrics and Computational Enhancement for Assessing Lupus Nephritis Activity
Source: Biosensors (Basel). 2025 Jan 11;15(1):39. doi: 10.3390/bios15010039 (PMC11763532; doi:10.3390/bios15010039)
Supplement: Supplementary file 1 [file biosensors-15-00039-s001.zip › Figure S2.pdf]

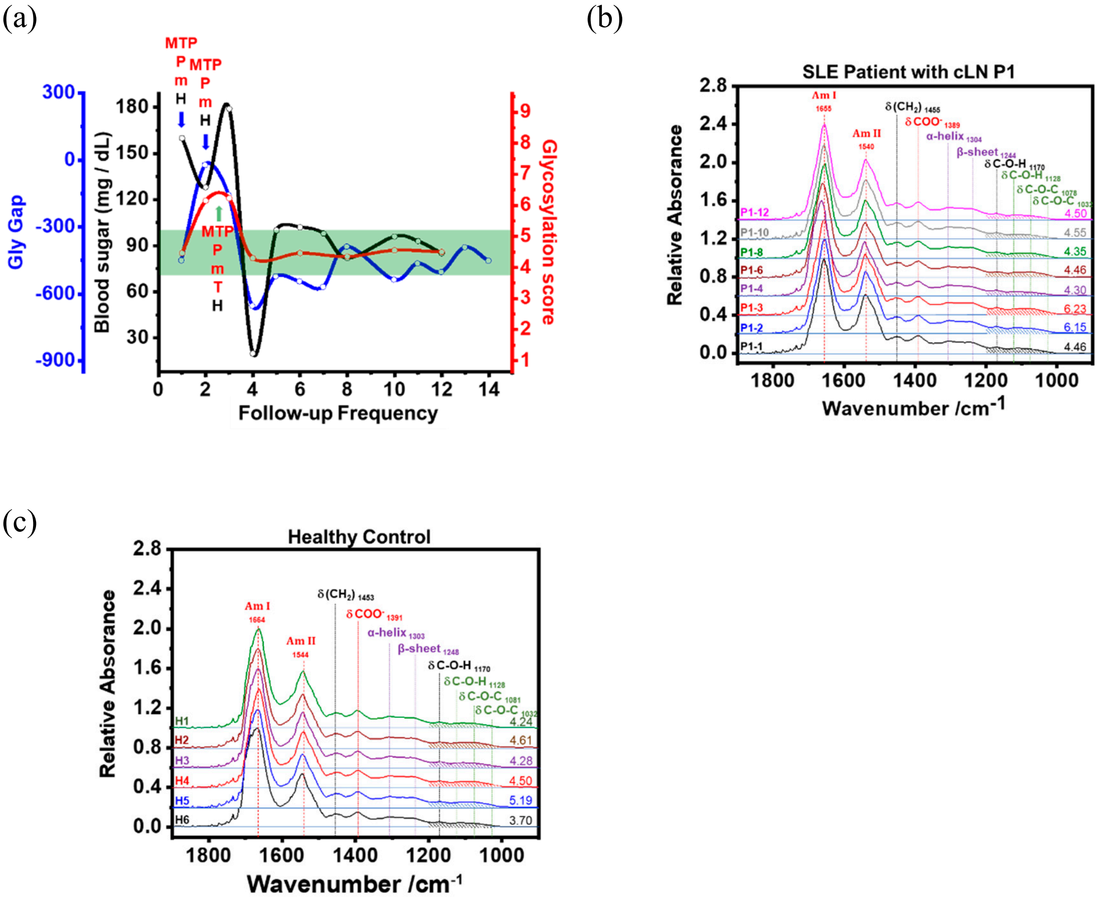

Figure S2. Correlation between IgG glycosylation and the Gly spectral index. **Panel (a)** illustrates the profiles for blood sugar levels (in black), the Gly spectral index (in blue), and the glycosylation level of IgG (in red), isolated from lupus patient P1 with acute cLN using fast protein liquid chromatography (FPLC). The red shading indicates the glycosylation score for IgG of a healthy control subject. **Panels (b)** and **(c)** present the glycosylation scores of IgG isolated from serial serum samples of lupus patient P1 and healthy control subjects (H1-H6), respectively, also obtained via FPLC. The glycosylation score, indicative of IR absorbance, is determined by integrating the absorption in the spectral range of 1200-1000 cm^{-1}, corresponding to the area under the spectral curve.
