# Supplementary material for: Developing a Label-Free Infrared Spectroscopic Analysis with Chemometrics and Computational Enhancement for Assessing Lupus Nephritis Activity
Source: Biosensors (Basel). 2025 Jan 11;15(1):39. doi: 10.3390/bios15010039 (PMC11763532; doi:10.3390/bios15010039)
Supplement: Supplementary file 1 [file biosensors-15-00039-s001.zip › Figure S3.pdf]

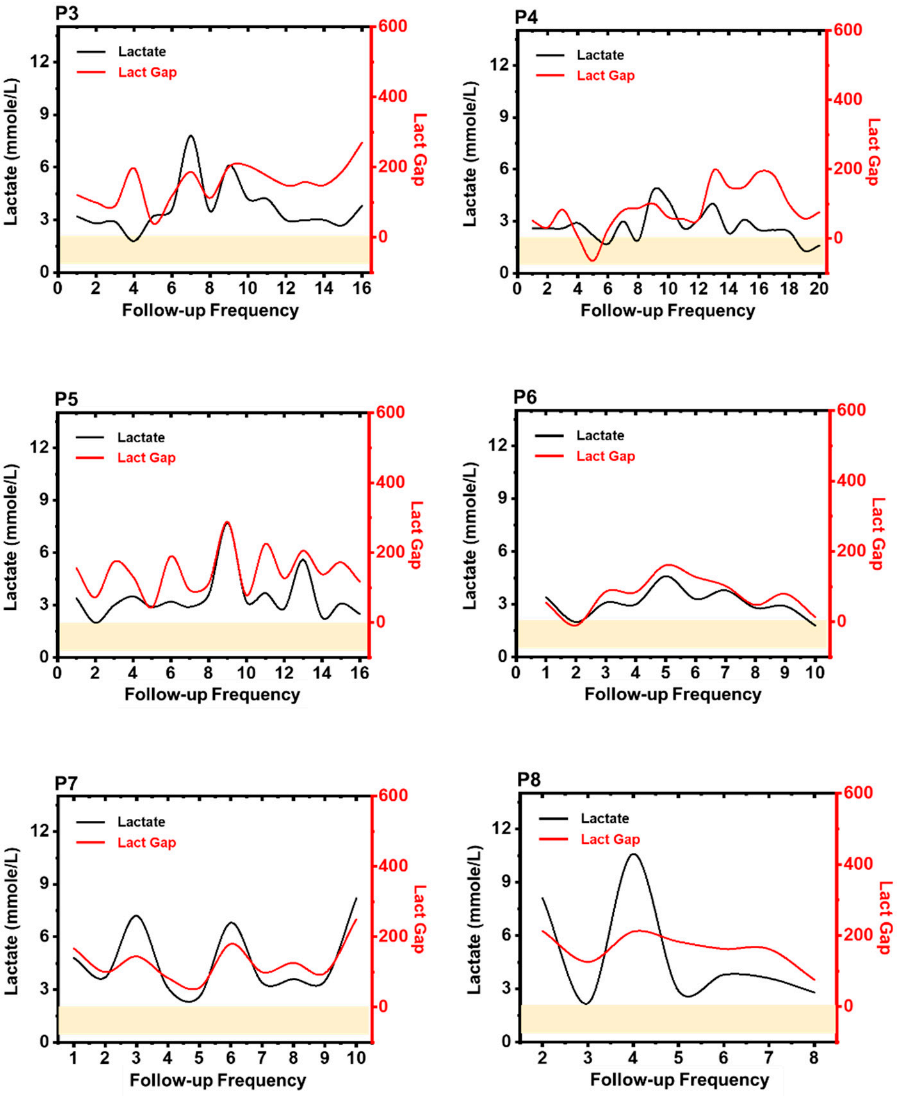

Figure S3. The figures present the correlation between the Lact spectral index and serum lactate levels throughout the treatment of cLN patients P3-P8. The consistency between the Lact spectral index and serum lactate levels, measured using a portable lactate meter, is demonstrated.
