# Supplementary material for: Developing a Label-Free Infrared Spectroscopic Analysis with Chemometrics and Computational Enhancement for Assessing Lupus Nephritis Activity
Source: Biosensors (Basel). 2025 Jan 11;15(1):39. doi: 10.3390/bios15010039 (PMC11763532; doi:10.3390/bios15010039)
Supplement: Supplementary file 1 [file biosensors-15-00039-s001.zip › Table S1.pdf]

Table S1. Concentrations of clinical biomarkers (UTP, UPCR, Scr, eGFR, serum albumin) for acute cLN patients P1-P4 and chronic cLN P5-P8 at each follow-up.

Group	Patient	Follow-up frequency	Follow-up time (day)	UTP (mg/dL)	UPCR (mg/g)	Scr (mg/dL)	eGFR (mg/min/1.73m^2)	Albumin (g/dL)
Acute cLN	P1	P1-1	0	1366.8	15.0	1.35	48.9	2.74
		P1-2	18	274.8	6.0	0.87	76.0	2.22
		P1-3	92	304.8	3.3	0.49	135.7	3.12
		P1-4	330	23.3	1.7	0.65	101.7	4.5
		P1-5	477	23.6	0.1	0.62	106.6	4.59
		P1-6	580	7.1	0.2	0.59	112.8	4.63
		P1-7	698	12.9	0.1	0.61	109.3	4.88
		P1-8	848	5.5	0.1	0.53	159.2	4.45
		P1-9	939	18.4	0.1	0.54	153.2	4.75
		P1-10	1016	11.3	0.1	0.57	145.5	4.81
		P1-11	1100	17.3	0.1	0.64	127.1	4.75
		P1-12	1191	4.2	0.1	0.61	133.9	4.63
Acute cLN	P2	P2-1	0	233.3	3.7	0.45	131.2	3.19
		P2-2	229	31.1	0.2	0.46	129.1	4.55
		P2-3	320	19	0.1	0.34	176.1	4.25
		P2-4	446	11.8	0.0	0.48	126.7	4.26
		P2-5	486	12.2	0.1	0.41	149.1	4.56
		P2-6	605	17.1	0.4	0.4	155.2	4.32
		P2-7	768	27	0.5	0.46	136.6	4.78
Acute cLN	P3	P3-1	0	820.5	15.0	0.98	63.1	2.83
		P3-2	10	712.6	13.3	1.14	54.5	2.87
		P3-3	36	950.8	6.4	0.97	63.9	2.82
		P3-4	53	441.8	9.3	0.89	69.6	3.94
		P3-5	81	517.8	10.8	0.76	81.0	3.28
		P3-6	120	255.2	8.9	0.6	103.3	3.73
		P3-7	137	547.7	9.5	0.6	103.3	3.76
		P3-8	154	544.2	8.3	0.76	81.2	3.5
		P3-9	172	495.7	5.9	0.94	65.5	4.13
		P3-10	197	666.7	10.3	0.65	94.7	3.61
		P3-11	225	859.5	5.1	0.7	87.9	3.52
		P3-12	274	355.6	2.3	0.9	68.4	4.07
		P3-13	361	306.5	1.9	0.95	65.3	4.28
		P3-14	438	675.6	7.1	0.79	79.0	3.93
Acute cLN	P4	P4-1	0	220.3	7.4	5.5	11.0	3.11
		P4-2	12	140	3.8	2.16	28.1	2.34
		P4-3	15	2.6	0.1	2.17	28.0	3.36
		P4-4	135	212.7	3.3	1.72	35.3	3.5
		P4-5	233	102.3	2.0	1.46	42.0	3.9
		P4-6	268	61.2	0.6	1.7	35.9	4.09

		P4-7	345	55.1	0.8	1.25	49.1	4.43
		P4-8	366	185.1	1.6	1.29	47.6	4.28
		P4-9	394	137.3	2.1	1.25	49.1	4.26
		P4-10	457	155.9	2.4	1.35	45.6	4.1
		P4-11	495	145	1.6	1.69	36.4	4.01
		P4-12	534	90.9	1.4	1.58	39.0	4.36
		P4-13	576	83.9	1.0	1.77	34.9	4.12
		P4-14	639	107.1	1.1	1.72	35.8	4.17
		P4-15	695	64.4	0.7	2.08	29.7	4.55
		P4-16	769	67.7	0.9	1.92	32.1	4.41
		P4-17	811	20.8	0.2	1.85	33.4	4.42
		P4-18	825	42.9	0.4	1.97	31.3	4.1
Chronic cLN	P5	P5-1	0	329.7	3.2	1.09	65.1	4.18
		P5-2	33	233.8	3.3	0.87	84.4	3.76
		P5-3	41	250.9	3.7	0.9	81.2	3.63
		P5-4	221	278.1	4.8	1.11	63.5	3.46
		P5-5	336	247.9	2.6	1.31	52.3	3.72
		P5-6	392	415.2	5.3	1.41	48	3.66
		P5-7	413	416.9	4.9	1.25	55.1	3.77
		P5-8	567	339.3	3.9	1.75	37.2	3.73
		P5-9	584	646.1	6.0	1.41	47.8	3.56
		P5-10	609	352.3	4.4	1.78	36.5	3.81
		P5-11	665	268.9	3.5	1.68	39	3.97
		P5-12	840	329.4	4.9	2.4	25.7	3.9
		P5-13	879	313	4.7	2.28	27	4.31
		P5-14	938	229.7	3.1	2.52	24.3	4.27
		P5-15	994	256.5	2.1	2.76	21.8	4.27
Chronic cLN	P6	P6-1	0	298.8	1.5	0.51	132.0	4.21
		P6-2	14	142.9	1.2	0.49	139.3	3.95
		P6-3	148	76.6	1.4	0.63	108.2	4.09
		P6-4	232	53.7	0.8	0.58	117.6	4.32
		P6-5	368	447.1	1.7	0.53	129.0	4.20
		P6-6	452	58	1.3	0.57	120.1	4.33
		P6-7	508	81.6	1.0	0.6	138.4	4.1
		P6-8	648	101.2	1.2	0.58	143.3	4.09
Chronic cLN	P7	P7-1	0	148.3	1.9	0.65	114.3	3.46
		P7-2	7	233	2.5	0.67	110.4	3.84
		P7-3	49	96.6	2.0	0.77	93.9	3.9
		P7-4	77	63.1	3.4	0.76	95.3	4.28
		P7-5	161	77.5	0.9	0.72	101.3	4.22
		P7-6	217	60.6	0.7	0.73	99.6	3.96
		P7-7	378	53.6	1.0	0.63	117.7	4.05

		P7-8	521	47.9	0.5	0.64	115.3	3.96
		P7-9	689	85.4	0.6	0.77	92.8	4.12
Chronic cLN	P8	P8-1	0	27.3	1.1	0.76	100.4	4.11
		P8-2	364	29.6	2.5	0.61	128.3	3.87
		P8-3	602	91.6	5.3	0.69	110.7	3.88
		P8-4	644	80.2	5.9	0.73	103.7	4.05
		P8-5	735	240.5	6.8	0.62	124.9	3.89
		P8-6	924	70.1	5.6	0.75	99.8	3.68
		P8-7	990	220.1	5.9	0.81	91.2	3.5
